# Seasonal Variation in Myocardial Infarction Hospitalisations and Ischaemic Heart Disease Deaths in New Zealand

**DOI:** 10.7759/cureus.81005

**Published:** 2025-03-22

**Authors:** David Bassett, Alistair J Woodward, Joshua Read

**Affiliations:** 1 Radiology, Tauranga Hospital, Tauranga, NZL; 2 Epidemiology and Biostatistics, The University of Auckland, Auckland, NZL; 3 Ophthalmology, Rotorua Eye Clinic, Rotorua, NZL

**Keywords:** death, hospitalisation, incidence, ischaemic heart disease, mortality, multi-centre, myocardial infarction, new zealand, seasonality, seasonal variation

## Abstract

Background

Seasonal variation in the incidence of myocardial infarction (MI) has been reported, but the literature is conflicting regarding its magnitude and geographical differences. This study examined the seasonal variation in MI incidence and ischaemic heart disease (IHD) mortality across three New Zealand cities: Auckland, Wellington, and Christchurch.

Methods

This multi-centre retrospective cohort study used New Zealand Ministry of Health data from 2005 to 2014. Poisson regression analysis was conducted, controlling for age, city, and secular trends. Event counts were grouped by season and multivariate models calculated seasonal event ratios with summer as the reference season.

Results

From 2005 to 2014, there were 42,846 MI hospitalisations and 15,466 deaths from IHD. Compared to summer, winter had 1,278 additional MI hospitalisations and 1,100 more IHD-related deaths. Winter-to-summer event ratios were 1.29 (95% confidence interval (CI): 1.15-1.45, p<0.001, χ²=900.84) for MI hospitalisations and 1.35 (95% CI: 1.28-1.43, p<0.001, χ²=61.10) for IHD mortality. The seasonal effect was most pronounced among those aged 80 years and older. No statistically significant differences were observed between cities, genders, ethnicities, or socioeconomic groups.

Conclusion

MI incidence varies by season, peaking during the winter and declining in summer. Given the substantial seasonal burden, targeted interventions such as public awareness campaigns, influenza vaccinations, improved indoor heating, and energy subsidies for vulnerable populations may help reduce winter cardiovascular disease (CVD) risk.

## Introduction

Seasonal variation in mortality and morbidity is well-documented, with rates peaking in winter and declining in summer [1-6]. While respiratory infections contribute to this trend, they are not the sole cause. Cardiovascular diseases (CVDs), particularly myocardial infarction (MI), also exhibit seasonal patterns in both incidence and mortality [6-11]. The underlying mechanisms remain uncertain, but several contributing factors have been proposed. Blood pressure and serum cholesterol tend to rise during winter [11-15]. This is hypothesized to be due to cold-induced vascular resistance, reduced physical activity, lower vitamin D levels, and heightened catecholamine release. Physical inactivity during colder months further worsens body weight, metabolic profiles, and cardiovascular fitness, compounding MI risk. Additionally, the higher prevalence of respiratory infections, particularly influenza, during winter further increases MI incidence [16,17].

As CVD is the leading cause of mortality globally, even small fluctuations in incidence can have significant public health implications [18]. Accordingly, understanding and mitigating the seasonal increases in CVD could substantially improve population health. However, the extent of seasonal variation in ischaemic heart disease (IHD) across different cities and climates remains unclear, and methodological differences make it difficult to compare results. Identifying regional differences may offer insights into environmental contributors and facilitate targeted interventions.

New Zealand provides a unique setting to explore these variations. Its three major cities, Auckland, Wellington, and Christchurch, share similar population demographics and healthcare access but differ in climate [19]. Auckland has a relatively stable, warm climate. Christchurch experiences pronounced seasonal temperature changes. Wellington has the mildest seasonal variability. We hypothesized that Christchurch would exhibit the greatest seasonal variation due to its larger temperature fluctuations. However, other climatic factors, such as Wellington’s wind chill, may also influence seasonal cardiovascular risk.

The primary aim of this study was to quantify the extent of seasonal variation in MI hospitalisations and IHD mortality across Auckland, Wellington, and Christchurch. The secondary aims were to evaluate how these seasonal trends vary by age group and geographic region. Specifically, we sought to determine whether elderly individuals experience a more pronounced seasonality and whether differences in climate contribute to regional variation.

This article was previously published as an abstract in the Heart, Lung and Circulation Journal in 2022.

## Materials and methods

Study design

A retrospective observational study was conducted using data from the New Zealand Ministry of Health's National Minimum Dataset (hospital events) and Mortality Collections [20]. Each dataset was analysed individually.

Study population

Hospitalisations due to MI (International Classification of Diseases, 10th Revision (ICD-10) codes I21-I22) and deaths attributed to IHD (ICD-10 codes I20-I25) were extracted for Auckland, Wellington, and Christchurch from December 2005 to November 2014 [21]. ICD-10 codes I21-I22 were chosen for MI because they specifically capture acute MI events, distinguishing them from chronic or past infarctions, while I20-I25 encompass the broader spectrum of IHD, including both acute and chronic presentations.

Inclusion and exclusion criteria

To maintain event independence, only the first MI hospitalisation per individual was included in the analysis. This approach ensures that recurrent admissions for the same MI event do not artificially inflate hospitalisation counts. However, it may lead to an underestimation of the total MI burden, as subsequent infarctions within the study period are excluded.

Ethics

Ethics approval was granted by the Northern Auckland Health and Disability Ethics Committee (MEC/07/19/EXP/AM14).

Statistical analysis

Cities were defined by District Health Board (DHB) regions as outlined in Table 1. Individual hospitalisation and mortality records were linked to a DHB using healthcare facility codes or Public Health Organisation enrollment data. Age, gender, and ethnicity data were recorded for each event. Event counts were grouped by season based on admission date with autumn beginning on 1st March, winter 1st June, spring 1st September and summer 1st December. Differences in the number of days between seasons were adjusted for. Poisson regression models were used to calculate seasonal event ratios, using summer as the reference season, and statistical significance was assessed using Wald tests. The chi-square values reported correspond to Wald tests of the regression coefficients, each with one degree of freedom. Multivariate models adjusted for age, city, and secular trends which were controlled for by including a year variable in the regression model to account for long-term changes in MI and IHD incidence over time. The resulting terms were seasonal event rate ~ season + period + city + age + age x season. Age, city, gender and ethnicity interaction terms were tested but only the age-season interaction term was statistically significant, hence its inclusion. To maintain sufficiently large study numbers to permit statistical testing, the study population was divided into those aged less than 80 years and those aged 80 years and over (as this age was near the median age in both the hospitalisation and mortality datasets).

**Table 1:** The district health boards included in each region

Region	District Health Board
Auckland	Waitemata, Auckland and Counties Manukau
Wellington	Hutt Valley, Capital and Coast
Canterbury	Canterbury

## Results

Table 2 records the subjects’ basic demographics. There were 42,846 hospitalisations and 15,466 deaths from IHD from December 2005 to November 2014 in Auckland, Wellington and Christchurch. The mean age of those dying from IHD was 15.3 years higher than those hospitalised for MI. This suggests that older individuals are more likely to die from IHD rather than be hospitalised, possibly due to increased comorbidities and higher frailty. Patients hospitalised were more likely to be male and Māori than those who passed away from IHD. Auckland had the greatest event counts, followed by Christchurch then Wellington. When adjusted for population size using the New Zealand 2013 Census [22], MI hospitalisation rates per 1,000 residents were highest in Wellington (20.89), followed by Christchurch (18.45) and Auckland (16.14). IHD mortality per 1,000 residents was highest in Christchurch (7.82), followed by Wellington (6.29) and Auckland (5.85).

**Table 2:** Basic demographics of myocardial infarction patients.

	Hospitalisations	Mortality
Total	42,846	15,466
Age (Mean ± Standard Deviation)	67.8 ± 14.0	83.1 ± 10.3
Māori Ethnicity, n (% of total)	3,352 (7.9)	675 (4.3)
Female, n (% of total)	15,202 (35.6)	7,946 (51.4)
Auckland, n (% of total)	22,850 (53.6)	8,282 (53.5)
Wellington, n (% of total)	9,846 (23.1)	2,965 (19.2)
Christchurch, n (% of total)	9,950 (23.3)	4,219 (27.3)

Table 3 shows the event ratios for each season. Seasonality is evident, with winter having 1,278 more MI hospitalisations and 1,100 more IHD mortalities than summer. Notably, while MI hospitalisation event ratios for spring and autumn were nearly equal, IHD mortality remained higher in spring than in autumn. This suggests a lagging effect of winter’s increased cardiovascular burden, where vulnerable patients who experience winter-related cardiovascular stressors may succumb in early spring. Additionally, prolonged respiratory infections, delayed health-seeking behaviour, or residual physiological stress from winter may contribute to sustained excess mortality into spring.

**Table 3:** Myocardial infarction hospitalisation and ischaemic heart disease mortality event ratios by season. The reference season was summer.

Season	MI Hospitalisation Event Ratio (95% CI)	p-value	Chi-square	IHD Mortality Event Ratio (95% CI)	p-value	Chi-square
Summer	1.00	-	-	1.00	-	-
Autumn	1.06 (1.03-1.09)	<0.001	14.49	1.09 (1.04-1.14)	<0.001	11.41
Winter	1.12 (1.09-1.15)	<0.001	54.82	1.31 (1.25-1.37)	<0.001	77.81
Spring	1.05 (1.03-1.08)	<0.001	14.63	1.19 (1.13-1.24)	<0.001	38.43

Table 4 presents the event ratios stratified by city. While Auckland and Christchurch exhibited significant seasonal variation in MI hospitalisations, Wellington showed no statistically significant effect (p > 0.05 for all seasons), reflected in lower chi-square values. This may be due to Wellington’s smaller population and its milder, more stable climate, which has the least seasonal temperature variability among the three cities. Differences in healthcare-seeking behaviour could also contribute to this pattern.

**Table 4:** Myocardial infarction (MI) hospitalisation and ischaemic heart disease (IHD) mortality seasonal event ratios stratified by city. The reference season was summer.

	MI Hospitalisations Event Ratio (95% CI)	p-value	Chi-square	IHD Mortality Event Ratio (95% CI)	p-value	Chi-square
Auckland						
Summer	1.00	-	-	1.00	-	-
Autumn	1.06 (1.02-1.10)	0.003	8.15	1.08 (1.01-1.15)	0.023	46.4
Winter	1.13 (1.09-1.18)	<0.001	28.34	1.34 (1.26-1.43)	<0.001	45.54
Spring	1.06 (1.02-1.10)	0.004	8.15	1.21 (1.14-1.29)	<0.001	24.82
Wellington						
Summer	1.00	-	-	1.00	-	-
Autumn	1.04 (0.98-1.10)	0.212	1.64	1.14 (1.02-1.27)	0.021	4.22
Winter	1.02 (0.96-1.08)	0.528	0.42	1.37 (1.23-1.52)	<0.001	18.11
Spring	1.03 (0.97-1.09)	0.391	0.93	1.24 (1.12-1.38)	<0.001	10.52
Christchurch						
Summer	1.00	-	-	1.00	-	-
Autumn	1.08 (1.02-1.14)	0.009	6.32	1.09 (1.00-1.19)	0.063	3.16
Winter	1.19 (1.13-1.26)	<0.001	27.51	1.20 (1.10-1.31)	<0.001	11.58
Spring	1.08 (1.02-1.14)	0.012	6.32	1.11 (1.02-1.21)	<0.001	4.64

For IHD mortality, seasonal variation was observed in all cities except for autumn in Christchurch, where the event ratio (1.09, 95% confidence interval (CI): 1.00-1.19, p = 0.063) did not reach statistical significance. This distinguishes it from other seasons, which all showed significant increases in mortality compared to summer.

Overall, Auckland and Christchurch displayed clear seasonal variation in both MI hospitalisations and IHD mortality, whereas Wellington exhibited seasonal variation only in IHD mortality, with no significant seasonality in MI hospitalisations.

Table 5 presents the event ratios stratified by age group. Seasonality in MI hospitalisations and IHD mortality was observed in both age groups, though the effect was more pronounced in those aged 80 and over.

**Table 5:** Myocardial infarction (MI) hospitalisation and ischaemic heart disease (IHD) mortality seasonal event ratios stratified by age group. The reference season was summer.

Age Group	MI Hospitalisations Event Ratio (95% CI)	p-value	Chi-square	IHD Mortality Event Ratio (95% CI)	p-value	Chi-square
Under 80						
Summer	1.00	-	-	1.00	-	-
Autumn	1.03 (1.00-1.07)	0.043	2.74	1.01 (0.92-1.10)	0.903	0.47
Winter	1.07 (1.03-1.10)	<0.001	14.36	1.20 (1.10-1.31)	<0.001	11.58
Spring	1.04 (1.01-1.07)	0.012	6.57	1.09 (1.00-1.19)	0.021	3.16
80 and Over						
Summer	1.00	-	-	1.00	-	-
Autumn	1.14 (1.08-1.21)	<0.001	15.61	1.13 (1.07-1.19)	<0.001	15.94
Winter	1.30 (1.23-1.38)	<0.001	47.01	1.35 (1.28-1.43)	<0.001	61.51
Spring	1.10 (1.04-1.16)	0.002	9.69	1.23 (1.16-1.30)	<0.001	33.60

For IHD mortality, all seasons showed significant increases in mortality compared to summer, except for autumn in those under 80 (event ratio: 1.01, 95% CI: 0.92-1.10, p = 0.903), which was not statistically significant. This contrasts with the 80+ group, where autumn mortality remained elevated (event ratio: 1.13, 95% CI: 1.07-1.19, p < 0.001).

The more pronounced seasonal pattern in the 80+ group may be explained by a combination of physiological and environmental factors. Older individuals tend to have greater frailty, making them more vulnerable to seasonal stressors. Additionally, comorbidities are more prevalent in the elderly and could exacerbate winter-related cardiovascular events.

Table 6 presents the age and city-adjusted event ratios. Seasonality was evident, with significantly higher event rates in colder months (MI hospitalisation event ratio: 1.29, 95% CI: 1.15-1.45, p < 0.001, χ² = 29.94 and IHD mortality event ratio: 1.35, 95% CI: 1.28-1.43, p < 0.001, χ² = 90.42). For autumn MI hospitalisations (event ratio: 1.13, 95% CI: 1.00-1.27, p = 0.0496, χ² = 16.93), the p-value is borderline significant, warranting cautious interpretation.

**Table 6:** MI hospitalisation and IHD mortality event ratios by season, adjusted for age and city. The reference season was summer. MI: myocardial infarction; IHD: ischaemic heart disease

Season	MI Hospitalisation Event Ratio (95% CI)	p-value	Chi-square	IHD Mortality Event Ratio (95% CI)	p-value	Chi-square
Summer	1.00	-	-	1.00	-	-
Autumn	1.13 (1.00-1.27)	0.0496	16.93	1.11 (1.05-1.18)	<0.001	28.24
Winter	1.29 (1.15-1.45)	<0.001	29.94	1.35 (1.28-1.43)	<0.001	90.42
Spring	1.10 (0.97-1.24)	0.136	14.16	1.25 (1.18-1.31)	<0.001	72.14

## Discussion

This study confirmed a clear seasonal variation in MI hospitalisations and IHD mortality in New Zealand, with the lowest number of events occurring in summer. This pattern was particularly pronounced among older individuals, especially in Auckland and Christchurch, consistent with research from the UK, Switzerland, Japan, and Australia [6-11]. The greater susceptibility of older adults to seasonal fluctuations has also been observed in follow-up research from the Whitehall study and in Melbourne [6,10]. Despite Auckland’s climate being similar to Melbourne’s, we observed a 13% increase in IHD admissions in winter compared to summer, whereas a Melbourne-based study reported a 22% rise in admissions during July compared to November [10].

A multi-city study from Japan also found diminished seasonality in cities with more stable climates [11], aligning with our observation of lower seasonal variation in Wellington. This suggests that environmental stability may help mitigate seasonal cardiovascular risk. Interestingly, while Wellington exhibited seasonality in IHD mortality, MI hospitalisations did not follow the same pattern. This discrepancy may be explained by its milder climate, with an average monthly temperature variation of 7.4°C compared to 8.5°C in Auckland and 11°C in Christchurch [19]. However, additional factors such as differences in healthcare-seeking behavior and variations in cardiovascular risk profiles may also play a role.

These findings highlight the need for further research into regional differences to better understand the underlying mechanisms driving seasonal patterns. Additionally, they underscore the importance of targeted winter interventions, for example, public awareness campaigns, improved access to flu vaccinations, and heating subsidies for at-risk populations, to mitigate the seasonal increase in cardiovascular events.

A key strength of this study is that all cities share similar demographics, socioeconomic status, and healthcare access. Additionally, the dataset used is large with an excellent capture rate. Finally, because hospitalisation and mortality data are collected at a national level, differences in data quality between different centres are minimised.

Weaknesses of this study include the staggered implementation of high-sensitivity troponin testing across regions, which may have led to inconsistent classification of MI cases over time. The use of high-sensitivity troponin leads to more patients being diagnosed with non-ST-elevation myocardial infarction (NSTEMI) [23]. The variation in the timing of implementation means that some regions may have had artificially elevated MI hospitalisation rates sooner than others, potentially biasing the seasonal analysis. As a result, subsequent seasons following its introduction may have shown artificially higher event rates. Another limitation of this study is the lack of data on seasonal population changes. Almost twice as many tourists visited New Zealand during summer than winter (approximately 600,000 more), a significant amount given New Zealand’s population is 5,348,600 (as of September 2024) [24,25]. However, it is unclear whether these visitors significantly contribute to seasonal CVD trends. If the majority of tourists are younger and healthier, their impact on hospitalisation rates may be minimal, whereas if a substantial proportion consists of older individuals at higher cardiovascular risk, this could partially explain the observed seasonal differences. Finally, we were unable to adjust for comorbidities. Chronic conditions such as diabetes, hypertension, and chronic respiratory disease are known to influence cardiovascular risk and may contribute to seasonal variations in MI incidence and IHD mortality. Without individual-level data on comorbidities, it is difficult to determine whether the observed seasonal trends are driven primarily by external environmental factors (e.g., temperature changes, respiratory infections) or fluctuations in underlying health conditions. Future studies incorporating detailed patient-level health data would provide a clearer picture of how comorbidities interact with seasonal cardiovascular risk.

Unfortunately, we did not have access to more recent data. Reinvestigation of the seasonality of CVD in New Zealand during the COVID-19 pandemic could shed further light on two hypothesised explanatory mechanisms - concurrent respiratory infections and energy poverty. This is because the incidence of influenza dropped significantly during COVID-19 lockdowns [26]. Additionally, in recent years the cost of living in New Zealand has increased significantly, exacerbating energy poverty [27]. The effect of these factors on the seasonal incidence and mortality rates of MI in New Zealand is unknown.

## Conclusions

This study demonstrates clear seasonal variation in MI hospitalisations and IHD mortality in New Zealand, with summer having the lowest number of events. The effect was most pronounced in elderly individuals and in residents of Auckland and Christchurch, emphasising the need for targeted public health interventions during colder months. Potential strategies could include flu vaccination programs, heating subsidies for at-risk populations, and public awareness campaigns for early symptom recognition. These interventions may help mitigate the seasonal increase in MI and IHD mortality, particularly among older adults and vulnerable communities.

While Wellington exhibited less pronounced seasonal variation, the reason remains uncertain. Stable environmental conditions may play a role. Other possible explanatory factors such as differences in healthcare-seeking behaviours and comorbidities may also contribute. Further research is needed to determine the relative influence of temperature fluctuations, air pollution, and behavioural adaptations on seasonal CVD trends. Identifying these mechanisms will be crucial for refining preventive strategies and improving cardiovascular health outcomes year-round.
